# First-Principles Study of Point Defects in GaAs/AlAs Superlattice: the Phase Stability and the Effects on the Band Structure and Carrier Mobility

**DOI:** 10.1186/s11671-018-2719-7

**Published:** 2018-09-26

**Authors:** Ming Jiang, Haiyan Xiao, Shuming Peng, Liang Qiao, Guixia Yang, Zijiang Liu, Xiaotao Zu

**Affiliations:** 1 School of Physics, University of Electronic Science and Technology of China, Chengdu, 610054 China; 2 Institute of Nuclear Physics and Chemistry, Chinese Academy of Engineering Physics, Mianyang, 621900 China; 3 Department of Physics, Lanzhou City University, Lanzhou, 730070 China

**Keywords:** Hybrid density functional theory, Point defect, GaAs/AlAs superlattice, Electrical properties

## Abstract

Advanced semiconductor superlattices play important roles in critical future high-tech applications such as aerospace, high-energy physics, gravitational wave detection, astronomy, and nuclear related areas. Under such extreme conditions like high irradiative environments, these semiconductor superlattices tend to generate various defects that ultimately may result in the failure of the devices. However, in the superlattice like GaAs/AlAs, the phase stability and impact on the device performance of point defects are still not clear up to date. The present calculations show that in GaAs/AlAs superlattice, the antisite defects are energetically more favorable than vacancy and interstitial defects. The As_X_ (X = Al or Ga) and X_As_ defects always induce metallicity of GaAs/AlAs superlattice, and Ga_Al_ and Al_Ga_ antisite defects have slight effects on the electronic structure. For GaAs/AlAs superlattice with the interstitial or vacancy defects, significant reduction of band gap or induced metallicity is found. Further calculations show that the interstitial and vacancy defects reduce the electron mobility significantly, while the antisite defects have relatively smaller influences. The results advance the understanding of the radiation damage effects of the GaAs/AlAs superlattice, which thus provide guidance for designing highly stable and durable semiconductor superlattice based electronic and optoelectronics for extreme environment applications.

## Background

The superlattice (SL) is an artificial material consisting of alternating thin layers of two or more different components. The (GaAs)_n_/(AlAs)_m_ is one of the most important SL since the development of high electron mobility transistors (HEMT) and quantum cascade lasers (QCLs) a few decades ago [1–6]. Recently with the advances of film epitaxy and nanofabrication techniques, the (GaAs)_n_/(AlAs)_m_ based SLs and nanodevices with (n + m) ranging from 2 to 10 have demonstrated exciting physical properties related to luminescence and optical absorption, two-phonon absorption, and Raman as well as infrared spectra, which thus found promising applications in optoelectronics, sensing, LED, energy and laser related civilian and industrial areas [7–12]. Meanwhile, toward other critical high-tech applications such as aerospace, high-energy physics, gravitational wave detection, astronomy, space travel, nuclear and national security related areas, the semiconductor SLs and devices are exposed to different radiation environments, i.e., X-ray, neutrons, electrons, ions, etc., which may result in the generation of defects containing impurities, vacancies, interstitials, antisites, and complex of these. Since the semiconductor materials and related physical properties play an important role in operating and functioning these electronic devices and integrated circuits, small amounts of defects may drastically change their optical and transport properties, especially in multilayer systems [13].

The effects of foreign impurities or intrinsic defects on the semiconductor SLs and their component materials have been extensively investigated in the past decades. Zollo et al. have employed density functional theory (DFT) method to investigate the stability of point defects in GaAs, and found that the antisite defects were more favorable [14]. Kahaly et al. have studied GaAs/AlAs SL structure by DFT method and found the arsenic vacancy (V_As_) defect at and near the interface led to a conducting quasi 2-DEG between insulating dielectric arsenide [7]. Spasov et al. have studied the effects of nitrogen impurities on carrier transport and electron-hole recombination in GaAs/AlAs SL diodes [9]. They reported that the N impurities modified the energy of the electronic miniband and impeded electron diffusion through the SL miniband, which may lead to a strong radiative recombination of electron-hole pairs [9]. Wang et al. studied the inter-diffusion induced by the Zn impurity in GaAs/AlAs SL structures employing an *ab initio* molecular dynamics (AIMD) method [15]. Their results suggested that the Zn diffusion was assisted by the group-III elements, which were ejected into the interstitial channel and diffused rapidly, thereby disordering the superlattice [15]. Mitra and Stark found that the presence of vacancies enhanced the Ga/Al intermixing in GaAs/AlAs SL, resulting from the proposed two-atom ring mechanism of diffusion [16]. Recently, an AIMD simulation of radiation response of GaAs/AlAs SL has been carried out [17], in which the minimum energies for each atom to be permanently displaced from its lattice site have been determined, the pathways for defect generation have been provided, and the types of created defects have been identified. It revealed that the created Ga (or Al or As) Frenkel pair and As_Ga_-Ga_As_ antisite pair have profound effects on the density of state distribution and band structure of GaAs/AlAs SL [17].

So far, the stability of point defects in SL structure and the transport properties like carrier mobility still remain unknown. It is thus of vital importance to investigate how the presence of vacancy, interstitial and antisite defects influences the structural stability and electrical properties of GaAs/AlAs SL. In this study, the phase stability of single Ga (or Al or As) vacancy, single Ga (or Al or As) interstitial and single Ga_As_ (or Al_As_ or As_Ga_ or As_Al_) antisite defects have been studied. It is shown that the antisite defects are energetically more favorable than vacancy and interstitial defects. The band structures of these defective states have been investigated by the hybrid DFT method, which incorporates a portion of exact exchange from Hartree–Fock theory with the rest of the exchange-correlation energy from other sources (*ab initio* or empirical) [18], and is expected to offer a more accurate description of electronic structure of semiconductor materials than the standard DFT. In particular, the electron mobility has been predicted. It turns out the interstitial and vacancy defects reduce the electron mobility significantly, while the antisite defects have relatively smaller influences. This work will advance the understanding of the radiation damage effects of the semiconductor superlattice and provide guidance for designing highly stable and durable semiconductor superlattices-based electronic and optoelectronics for extreme environment applications.

## Methods

**Fig. 1:**
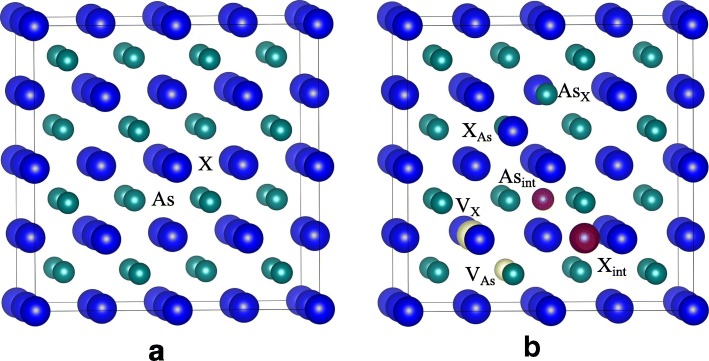
Schematic view of geometrical structures of **a** XAs (X = Ga or Al); **b** the defects in XAs. *V*_*X*_: (X = Ga, Al, or As) X vacancy; *X*_*int*_: X interstitial; *X*_*As*_: X occupying the As lattice site; *As*_*X*_: As occupying the X lattice site. The yellow and purple spheres represent the vacancy and interstitial defects, respectively

**Fig. 2:**
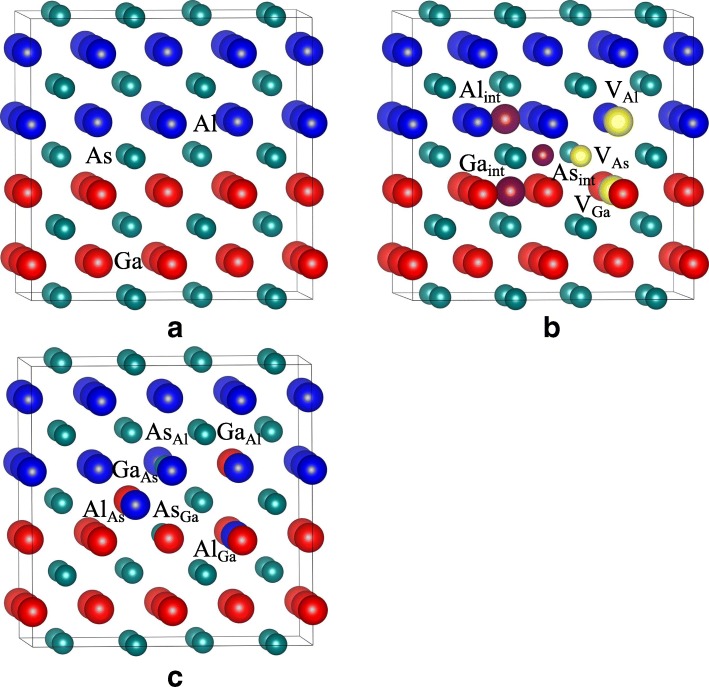
Schematic view of geometrical structures of **a** ideal GaAs/AlAs superlattice; **b** and **c** GaAs/AlAs superlattice with different point defects. *X*_*Y*_: (X, Y = Ga, Al, or As) X occupying the Y lattice site; *V*_*X*_: X vacancy; *X*_*int*_: X interstitial. The yellow and carmine spheres represent the vacancy and interstitial defects, respectively

In this study, the structural relaxations are carried out within the standard DFT framework and the band structures are calculated by the hybrid DFT in the framework of Heyd-Scuseria-Emzefhof (HSE) [19] based on the relaxed structures. All calculations are carried out employing Vienna *Ab Initio* Simulation Package (VASP) [20]. Projector augmented-wave pseudopotentials are used to describe the interaction between ions and electrons, and the exchange-correlation effects are treated using the local density approximation in the Ceperley-Alder parameterization [21]. The convergence criteria for total energies and forces are 10^−4^ eV and 10^−3^ eV/Å, respectively. The origin point group of AlAs and GaAs crystal is the *T*_*d*_ group of zinc blende, as shown in Fig. 1a. The illustration of considered point defects is provided in Fig. 1b. The GaAs/AlAs SL containing two monolayers of GaAs alternating with two monolayers of AlAs is considered in this study and the geometrical configuration is illustrated in Fig. 2, together with the considered point defects.

## Results and Discussion

### Ground State Properties of GaAs and AlAs

As shown in Table 1, the lattice constants of bulk GaAs and AlAs are determined to be 5.61 and 5.63 Å, respectively, which agree well with the experimental and other theoretical values [22–24]. It seems that the lattice mismatch between GaAs and AlAs is small, and the lattice constant of GaAs/AlAs SL is set to be the intermediate value of 5.62 Å. The bulk modulus is calculated by $B=\frac{1}{3}\left({C}_{11}+2{C}_{12}\right)$ [25], where the C_11_ and C_12_ represent the elastic constants. The bulk modulus of GaAs is calculated to be 76.3 GPa, which is close to the result of 76.5 GPa for AlAs. These results are in reasonable agreement with the theoretical and experimental data [22, 26, 27].

**Table 1:** The calculated and experimental ground state properties of bulk GaAs and AlAs. The a_0_ and B refer to the lattice constant and bulk modulus, respectively

	a_0_ (Å)	B (GPa)
GaAs		
Our Cal.	5.61	76.3
Other Cal.	5.61^a^	75.2^a^
Exp.	5.65^b^	76^c^
AlAs		
Our Cal.	5.63	76.5
Other Cal.	5.63^a^	75.1^a^
Exp.	5.66^d^	77.3^e^

^a^Ref. [22]

^b^Ref. [24]

^c^Ref. [27]

^d^Ref. [23]

^e^Ref. [26]

### The Defect Formation Energy in GaAs/AlAs Superlattice

For GaAs/AlAs SL and bulk states, the defect formation energy is calculated by ${E}_f={E}_{def}-{E}_{undef}+\sum \limits_i\Delta {n}_i{\mu}_i$ [28]. Here, *E*_*def*_ is the total energy of the defective simulation cell after relaxation, *E*_*undef *_ is the total energy of the relaxed ideal supercell, Δ*n*_*i*_ is the change in the number of species *i* (*i* = Ga, Al, or As), and *μ*_*i*_ is the chemical potential of species *i* [28].

**Fig. 3:**
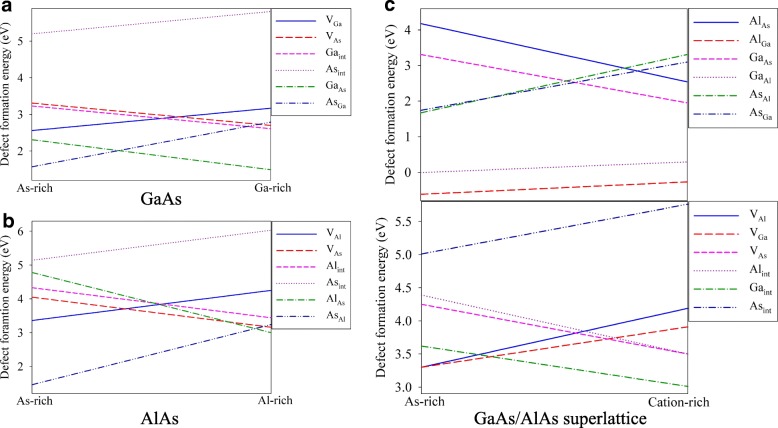
The defect formation energies under As-rich and cation-rich conditions in **a** GaAs, **b** AlAs and **c** GaAs/AlAs superlattice. *X*_*Y*_: (X, Y = Ga, Al, or As) X occupying the Y lattice site; *V*_*X*_: X vacancy; *X*_*int*_: X interstitial

For bulk XAs (X = Al or Ga), the chemical potentials of As and X obey the following constrains: ${\mu}_X\le {\mu}_X^{bulk}$, ${\mu}_{As}\le {\mu}_{As}^{bulk}$, and ${\mu}_{As}+{\mu}_X={\mu}_{XAs}^{bulk}$, where ${\mu}_X^{bulk}$, ${\mu}_{As}^{bulk}$, and ${\mu}_{XAs}^{bulk}$ correspond to the total energy of bulk X, bulk As and bulk XAs, respectively. The defect formation energies under X-rich condition, i.e., ${\mu}_X={\mu}_X^{bulk}$ and ${\mu}_{As}={\mu}_{XAs}^{bulk}-{\mu}_X^{bulk}$, and As-rich condition, i.e., ${\mu}_{As}={\mu}_{As}^{bulk}$ and ${\mu}_X={\mu}_{XAs}^{bulk}-{\mu}_{As}^{bulk}$, are summarized in Table 2. For GaAs, under As-rich conditions the As_Ga_ (As occupying the Ga lattice site) antisite defect is found to be the most energetically favorable, as indicated by the smallest formation energy of 1.57 eV. The next favorable defect is the Ga_As_ (Ga occupying the As lattice site) antisite defect, with the formation energy of 2.31 eV. The As interstitial (As_int_) has the largest formation energy of 5.20 eV, suggesting that it is more difficult to form than other considered point defects. Under Ga-rich conditions, the V_Ga_, As_int_ and As_Ga_ defects have larger formation energies, and the V_As_, Ga_int_ and Ga_As_ defects have smaller formation energies, as compared with the As-rich condition. Obviously, the defect stability depends on the chemical environment. As compared with GaAs, the defect formation energies in AlAs are generally larger, except the cases of As_int_ and As_X_ (X = Al or Ga) under As-rich conditions. The As_Al_ and Al_As_ antisite defects are determined to be the most favorable defect under As-rich and Al-rich conditions, respectively. Similar to the case of GaAs, the As_int_ is also unfavorable in AlAs. The defect formation energies under As-rich and X-rich (X = Ga or Al) conditions in bulk XAs are plotted in Fig. 3. Figure 3a shows that the As_Ga_ and Ga_As_ antisite defects are more favorable under As-rich and Ga-rich conditions, respectively. It is noted that the As_Al_ antisite defect is preferable in most cases (see Fig. 3b). Under Al-rich condition, the phase stability of Al_As_, V_As_ and As_Al_ defects are close to each other, as indicated by the formation energies of 3.0, 3.16 and 3.24 eV, respectively. Also, we find that in both GaAs and AlAs, the non-favorability of As_int_ is independent of the chemical environment. Zollo et al. carried out first-principles calculations on GaAs and their DFT results showed that the formation energies of As_Ga_ and Ga_As_ were smaller than those for vacancy and interstitial defects [14], which are consistent with our results.

**Table 2:** The calculated defect formation energies (eV) in bulk XAs (X = Al or Ga) under As-rich and X-rich conditions. The minimum values are indicated in italic

Defect	GaAs		AlAs	
	As-rich	Ga-rich	As-rich	Al-rich
V_X_	2.56	3.17	3.36	4.25
V_As_	3.31	2.7	4.05	3.16
X_int_	3.23	2.61	4.33	3.44
As_int_	5.20	5.81	5.14	6.03
X_As_	2.31	*1.49*	4.78	*3.0*
As_X_	*1.57*	2.79	*1.46*	3.24

*V*_*X*_: (X = Ga, Al, or As) X vacancy; *X*_*int*_: X interstitial; *X*_*As*_: X occupying the As lattice site; *As*_*X*_: As occupying the X lattice site

The *E*_*f*_ in GaAs/AlAs SL structure are also calculated under As-rich condition, i.e., ${\mu}_{As}={\mu}_{As}^{bulk}$, ${\mu}_{Al}={\mu}_{Al As}^{bulk}-{\mu}_{As}^{bulk}$, and ${\mu}_{Ga}={\mu}_{Ga As}^{bulk}-{\mu}_{As}^{bulk}$, and cation-rich condition, i.e., ${\mu}_{Al}={\mu}_{Al}^{bulk}$,${\mu}_{Ga}={\mu}_{Ga}^{bulk}$ and ${\mu}_{As}=\left({\mu}_{SL}^{bulk}-{n}_{Al}\times {\mu}_{Al}^{bulk}-{n}_{Ga}\times {\mu}_{Ga}^{bulk}\right)/{n}_{As}$, where *n*_*Al*_, *n*_*Ga*_, and *n*_*As*_ represent the number of Al, Ga and As atoms in the simulation cell, respectively. As shown in Table 3, the Al_Ga_ defect has negative formation energies, i.e., − 0.62 and − 0.27 eV under As-rich and cation-rich conditions, respectively, indicating that the formation of Al_Ga_ antisite defect is an exothermic process. As for Ga_Al_ defect, the formation energies are as small as − 0.01 eV under As-rich condition and 0.29 eV under cation-rich condition. Obviously, the formation of Al_Ga_ and Ga_Al_ antisite defects in the GaAs/AlAs SL structure are much easier than other point defects. Under As-rich condition, the formation energies of the second favorable defects of As_Ga_ and As_Al_ are determined to be 1.67 and 1.74 eV, respectively. For the interstitials, the phase stability both follows the trend of Ga_int_ > Al_int_ > As_int_ under As-rich and cation-rich conditions. The defect formation energies in GaAs/AlAs SL structure are also plotted in Fig. 3c. As compared with the bulk GaAs, the point defects in GaAs/AlAs SL are generally more difficult to form, except the case of As_int_ (see Fig. 3a, c). The formation energies of As_int_ in bulk GaAs are 5.20 and 5.81 eV under As-rich and Ga-rich conditions, which are slightly larger than the corresponding values of 5.01 and 5.76 eV in GaAs/AlAs SL. As shown in Fig. 3b and c, the stability of point defects in bulk AlAs and SL structure shows different character. The Al_As_ and As_int_ defects are more energetically favorable in GaAs/AlAs SL than bulk AlAs, whereas V_As_ defect is more preferable in bulk AlAs than SL structure. It is noticeable that under As-rich and Al-rich conditions, the formation energies of Al_int_ in bulk AlAs are comparable to that in GaAs/AlAs SL. Similar to the case of Al_int_, the V_Al_ defect in bulk AlAs and SL structure show similar favorability, as indicated by the comparable formation energies. In the case of As_Al_ defect, the formation energy under As-rich condition is smaller (1.46 eV) in SL structure, whereas under cation-rich condition, the value is smaller (3.10 eV) in bulk AlAs, suggesting that the stability of As_Al_ depends on the chemical environment.

**Table 3:** The calculated defect formation energies (eV) in GaAs/AlAs superlattice under cation-rich and As-rich conditions

Defect type	As-rich	Cation-rich
Antisite		
Ga_Al_	− 0.01	0.29
Ga_As_	3.31	1.95
Al_Ga_	− 0.62	− 0.27
Al_As_	4.18	2.54
As_Ga_	1.67	3.31
As_Al_	1.74	3.10
Vacancy		
V_Ga_	3.30	3.91
V_Al_	3.30	4.19
V_As_	4.25	3.50
Interstitial		
Ga_int_	3.62	3.01
Al_int_	4.39	3.50
As_int_	5.01	5.76

*X*_*Y*_: (X, Y = Ga, Al, or As) X occupying the Y lattice site; *V*_*X*_: X vacancy; *X*_*int*_: X interstitial

Comparing the defect stability in bulk AlAs, GaAs and GaAs/AlAs SL, we find that the antisite defects are always more preferable than vacancies and interstitials, especially for the cases of Ga_Al_ and Al_Ga_ in GaAs/AlAs SL. It is also noted that under As-rich and cation-rich conditions, the As_int_ defect is the most difficult to form in both bulk states and GaAs/AlAs SL structure.

### The Effects of Point Defects on the Band Structures of GaAs/AlAs Superlattice

#### The Pristine State of GaAs/AlAs Superlattice

**Fig. 4:**
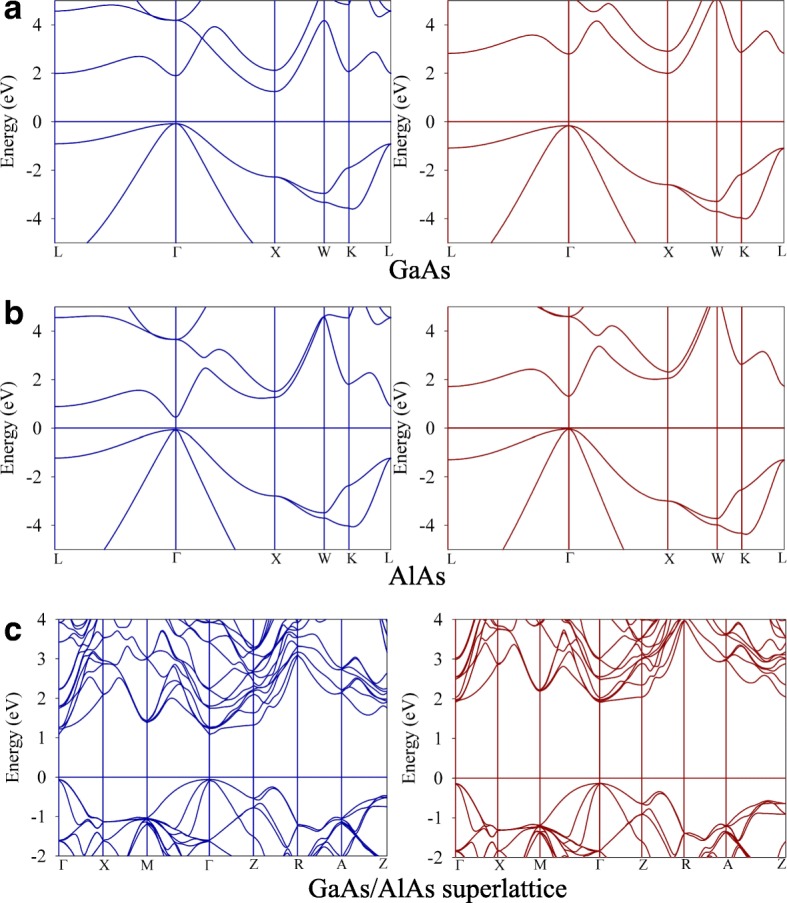
The band structures of **a** GaAs, **b** AlAs and **c** GaAs/AlAs superlattice. The hybrid DFT values are plotted in left-hand panels and the DFT results are plotted in the right-hand panels

The band gaps for bulk GaAs, AlAs and GaAs/AlAs SL are summarized in Table 4, and their band structures are presented in Fig. 4. The hybrid DFT calculations determine the direct band gap of GaAs to be 1.44 eV (see Fig. 4a), which agrees well with the experimental value of 1.52 eV [29] and other calculations [24]. By contrast, the standard DFT predicts a band gap value of 0.5 eV, which largely underestimates the band gap of GaAs. For AlAs, the band structure is of indirect character and the hybrid DFT band gap is 2.16 eV (see Fig. 4b), which is 0.85 eV larger than the DFT result and in good agreement with the experimental value of 2.22 eV [23]. As shown in Fig. 4c, the band gap of GaAs/AlAs SL is determined to be direct and it is consistent with the study of Botti et al., who found the band gap of (GaAs)_m_/(AlAs)_m_ SL (m ≥ 2) to be direct at the Γ point [3]. In our calculations, the direct band gap for GaAs/AlAs SL is determined to be 2.06 eV by hybrid DFT method, which is in agreement with the experimental value of 2.10 eV [30].

**Table 4:** The calculated band gap (eV) for bulk GaAs, AlAs, and GaAs/AlAs superlattice

	DFT	Hybrid DFT	Exp.
GaAs	0.50 (0.54^a^)	1.44 (1.36^b^)	1.52^c^
AlAs	1.31 (1.33^d^)	2.16 (2.24^e^)	2.22^f^
GaAs/AlAs SL	1.14 (1.16^g^)	2.06	2.09^h^

^a^Ref. [8]

^b^Ref. [29]

^c^Ref. [24]

^d^Ref. [40]

^e^Ref. [41]

^f^Ref. [23]

^g^Ref. [3]

^h^Ref. [30]

#### The Effects of Antisite Defects on the Band Structure of GaAs/AlAs Superlattice

**Fig. 5:**
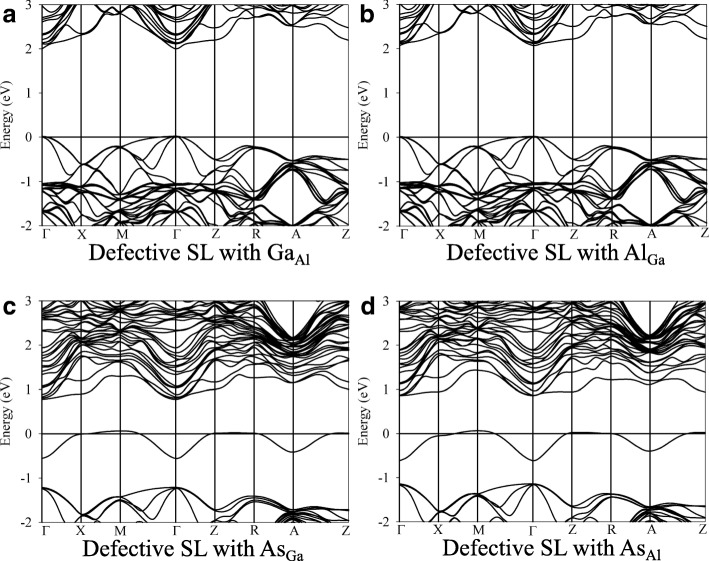
The band structures of defective GaAs/AlAs superlattice with different antisite defects. **a**: Ga occupying the Al lattice site; **b**: Al occupying the Ga lattice site; **c**: As occupying the Ga lattice site; **d**: As occupying the Al lattice site

In GaAs/AlAs SL structure, the Ga_Al_ and Al_Ga_ antisite defects are more energetically favorable than other point defects. As shown in Fig. 5a and b, the band structures of Ga_Al_ and Al_Ga_ defective states are very similar to that of the pristine state and the band gaps are determined to be 1.98 and 2.01 eV, respectively. This should be due to the fact that the Al and Ga chemical elements have similar valence electron configuration, i.e.,3s^2^3p^1^ for Al and 4s^2^4p^1^ for Ga, and no extra electrons or holes are introduced upon the formation of Ga_Al_ and Al_Ga_ antisite defects. The band structures for As_Ga_ and As_Al_ defective states are depicted in the Fig. 5c and d. It turns out that these two defects modify the band structure of GaAs/AlAs SL considerably. Both the As_Ga_ and As_Al_ antisite defects introduce extra electrons and act as n-type dopants. The impurity levels are found to be far from the valence bands and cross the fermi level, as shown in Fig. 5c and d. These deep defect levels may act as the recombination center for carriers.

**Fig. 6:**
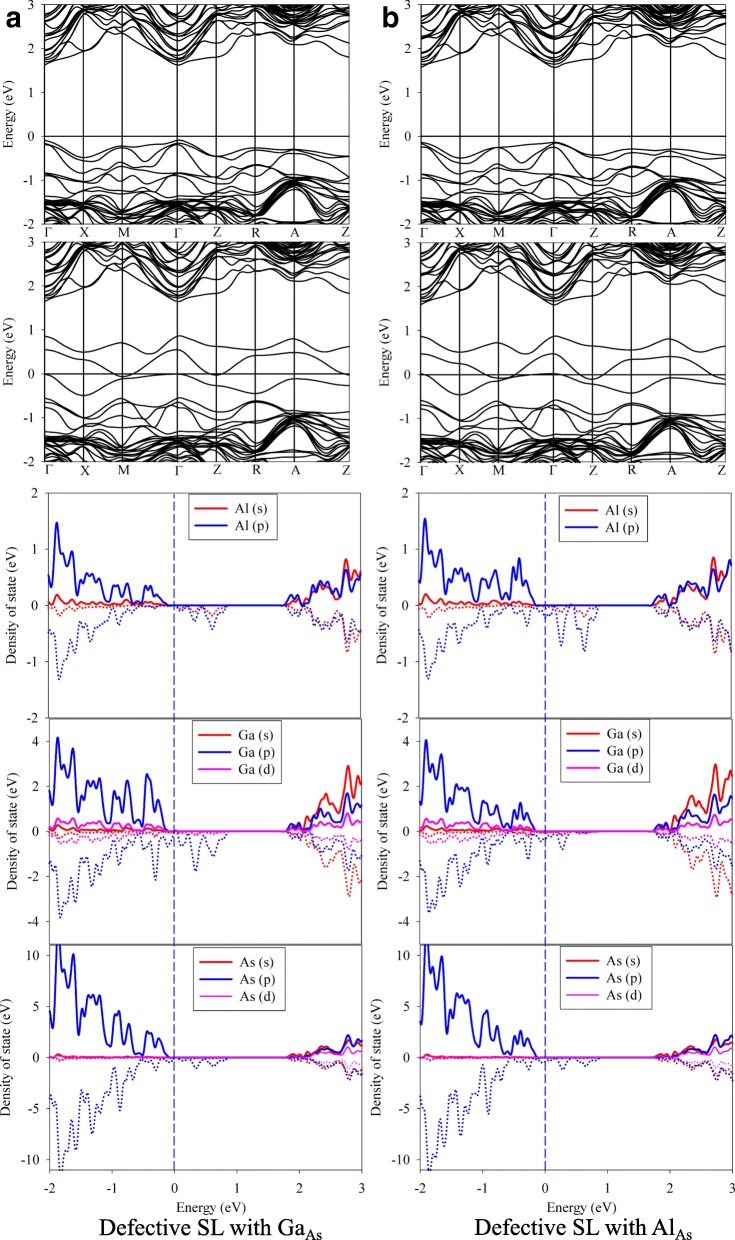
The band structures and partial density of state of defective GaAs/AlAs superlattice with **a** Ga_As_ and **b** Al_As_ antisite defects. *X*_*As*_ (X = Ga or Al) X occupying the As lattice site

Figure 6 presents the band structures and partial density of state (PDOS) of defective SL with Ga_As_ and Al_As_ defects. As shown in Fig. 6a, the band structure for Ga_As_ defective SL is of spin splitting character. In the spin-down subbands, the fermi level passes through the defect levels introduced by the Ga_As_ defect, indicative of half-metallic character of the defective SL. According to the definition of half-metallic gap [31], the band gap of Ga_As_ defective state is about 0.10 eV. As shown in the PDOS of the defective SL with Ga_As_, the spin-down subbands near the fermi level are mainly contributed by *p* partial waves. Due to the similar valence electron configurations between Ga and Al atoms, the calculated spin-up and spin-down band structures of Al_As_ defective state are determined (see Fig. 6b), and the band gap is calculated to be 0.15 eV. Overall, the Al_Ga_ and Ga_Al_ antisite defects have negligible effects on the electronic structure of GaAs/AlAs SL. It is also noted that the defective SL with As_Al_ and As_Ga_ defects show metallicity, while the defective SLs with Ga_As_ and Al_As_ are half-metallic.

#### The Effects of Vacancy Defects on the Band Structure of GaAs/AlAs Superlattice

**Fig. 7:**
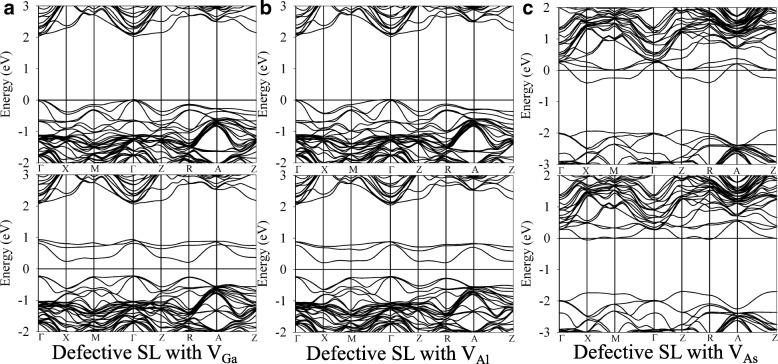
The band structures of defective GaAs/AlAs superlattice with **a** V_Ga_, **b** V_Al_ and **c** V_As_ vacancy defects. *V*_*X*_ (X = Ga, Al, or As) X vacancy

**Fig. 8:**
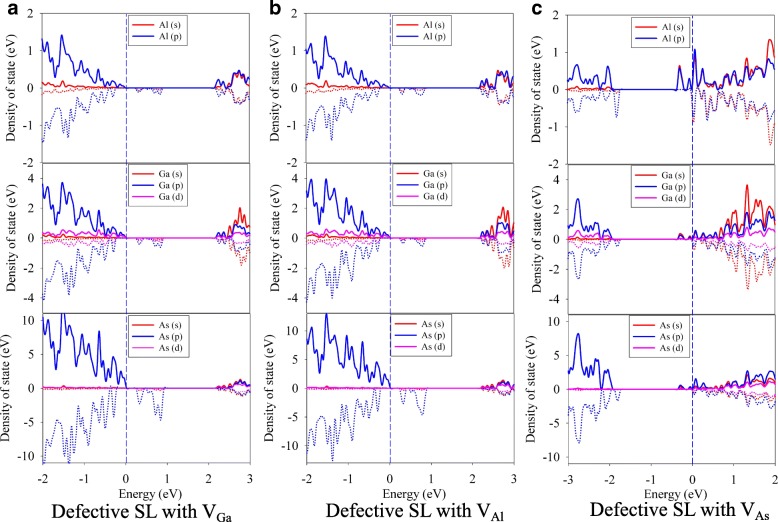
The partial density of state of defective GaAs/AlAs superlattice with **a** V_Ga_, **b** V_Al_ and **c** V_As_ vacancy defects. *V*_*X*_ (X = Ga, Al, or As) X vacancy

The band structures of SL structure with different vacancies are plotted in the Fig. 7, and their corresponding PDOS are depicted in Fig. 8. The spin splitting character of band structure is also found in the case of defective SL with V_Ga_ and V_Al_ defects, as shown in Fig. 7a and b. Indeed, removal of atoms from their original positions leaves four dangling bonds related to the *sp*^3^ orbitals. During the structural relaxation, the nearest atoms around the vacancy are equally displaced toward the empty lattice site, which results in site-symmetry defined by the tetragonal *D*_2*d*_ point group. The induced defect levels appear near the valence band and locate in the forbidden region of the GaAs/AlAs SL. The band gap is determined to be 0.47 and 0.44 eV for the SL with V_Ga_ and V_Al_ defects, respectively. As shown in the PDOS of defective SL with V_Ga_ and V_Al_ (see Fig. 8a and b), the main influence of the group-III vacancies is on the *p* states. As shown in Fig. 7c, the band structure of the defective SL with V_As_ defect splits into spin-up and spin-down parts, and the defect levels appear near the conduction band. Since the V_As_ defect acts as an n-type dopant, the fermi level shifts to higher energy and crosses the defect level edge. Kahaly et al. have investigated the electrical properties of the GaAs-AlAs heterointerfaces and found that V_As_ defect at the interface lead to quasi 2-DEG [7], which is consistent with our results. Our calculations show that the vacancies have different effects on the band structure of GaAs/AlAs SL, i.e., the V_As_ defect induces metallicity of GaAs/AlAs SL, and the V_Ga_ and V_Al_ defects reduce the band gap of SL structure significantly.

#### The Effects of Interstitial Defects on the Band Structure of GaAs/AlAs Superlattice

**Fig. 9:**
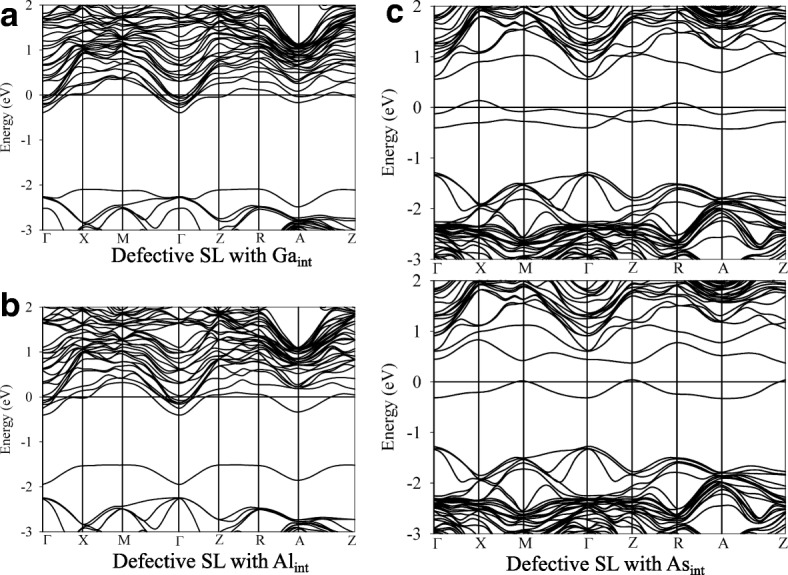
The band structures of defective GaAs/AlAs superlattice with **a** Ga_int_ defect, **b** Al_int_ defect and **c** As_int_ defect. *X*_*int*_ (X = Ga, Al, or As) X interstitial

Figure 9 presents the band structures of SL structure with interstitial defects. It is noted that the fermi level shifts to high energy and crosses the conduction band edge (see Fig. 9a and b), due to the fact that the group-III interstitials are donor-like defects. Consequently, the defective SLs with Ga_int_ and Al_int_ show metallic character. As shown in Fig. 9c, in the spin-up and spin-down parts of band structure, the impurity levels appear near the conduction band and the fermi level crosses the impurity level edge, indicating the induced metallicity of defective GaAs/AlAs SL with As_int_. Obviously, the interstitial defects significantly change the electronic structures of GaAs/AlAs SL and generally induce metallicity of defective SL structure.

Comparing the band structures and representative PDOS of the GaAs/AlAs SL with antisites, vacancies, and interstitials, we find that the defects modify the electronic structures considerably, except the cases of Ga_Al_ and Al_Ga_ antisite defects. Besides, the band gap narrowing and even metallicity are induced, which will influence the performance of GaAs/AlAs SL drastically.

### The Effects of Point Defects on the Electron Mobility of GaAs/AlAs Superlattice

The electron mobility at 0 K can be calculated from the equation *μ* = *eτ*/*m*^∗^, where *e* is the electron charge, *τ* is the relaxation time, and *m*^∗^ is the effective mass of carrier [32]. The electron effective masses can be evaluated from the curvature of the band structures via the relation ${m}^{\ast }={\mathrm{\hslash}}^2{\left(\frac{d^2\varepsilon }{dk^2}\right)}^{-1}$ [32], where ℏ is the reduced Planck constant, *k* is the wave vector, and *ε* is the energy of conduction band minimum. As shown in Fig. 4a and b, we obtain m^*^ = 0.057 *m*_e_ for GaAs and m^*^ = 0.19 *m*_e_ for AlAs, agreeing well with the experimental values of 0.057 *m*_e_ for GaAs [33] and 0.124 *m*_*e*_ for AlAs [34], where *m*_*e*_ is the static electron mass. The relaxation time for AlAs and GaAs is assumed to be 0.17 and 0.48 ps, respectively [35]. The electron mobility of GaAs and AlAs at 0 K are calculated to be 1.48  ×  10^4^cm^2^/Vs and 1.57  ×  10^3^ cm^2^/Vs, respectively, which is comparable to the experimental values of 0.94  ×  10^4^ cm^2^/Vs for GaAs [36] and 0.28  ×  10^3^ cm^2^/Vs for AlAs [37].

As shown in Table 5, the electron effective mass at the Г point (${m}_{\Gamma}^{\ast }$) is determined to be 0.113 *m*_*e*_ for the pristine GaAs/AlAs SL and the relaxation time *τ* is assumed to be 0.4 ps [38]. The electron mobility along the z direction, i.e., Γ-X direction in the Brillouin zone (*μ*_Γ − *X*_) is calculated to be 0.623  ×  10^4^ cm^2^/Vs for ideal GaAs/AlAs SL, which is comparable to the experimental value of 1.0  ×  10^4^ cm^2^/Vs [38]. As for the defective SL with antisite defects, the value of *μ*_Γ − *X*_ is comparable with that for the ideal SL, except for the cases of Ga_As_ and Al_As_ defects. The electron mobility along the Γ-X direction is determined to be 0.263  ×  10^4^ cm^2^/Vs and 0.311  ×  10^4^ cm^2^/Vs for Ga_As_ and Al_As_ defective states, respectively, which are much smaller than that for the ideal state. It is noted that the Ga_int_, Al_int_ and As_int_ defects also reduce the electron mobility significantly, as indicated by the values of 0.225  ×  10^4^ cm^2^/Vs for Ga_int_, 0.243  ×  10^4^ cm^2^/Vs for Al_int_ and 0.315  ×  10^4^ cm^2^/Vs for As_int_. As compared with antisite and interstitial defect, the vacancies have the most profound effects. For V_Ga_ and V_Al_ defects, the values of *μ*_Γ − *X*_ are about six times smaller than that of pristine state. The V_As_ defect also significantly decreases the electron mobility, as indicated by 0.127  ×  10^4^ cm^2^/Vs. Tanaka et al. have investigated the effects of electron irradiation on the electrical properties of GaAs/AlGaAs heterostructures and they found that the electron mobility was reduced at doses greater than 5 × 10^20^ cm^−2^ [10]. Especially, the defect creation in GaAs channel region, rather than n-AlGaAs layer, is thought to be the main cause of the mobility degradation [10]. Recently, it has been suggested that the electrons are possibly trapped by defects or impurity and produce metastable states accompanied by lattice relaxation [39]. Consequently, the electronic structure and carrier mobility of GaAs/AlAs SL are influenced significantly by the point defects. Therefore, it is necessary to enhance the radiation tolerance of GaAs/AlAs SL to improve its electronic performance under radiation environment.

**Table 5:** The band gap, electron effective mass at the Γ point (${m}_{\Gamma}^{\ast }$), and electron mobility along the z direction, i.e., Γ-X direction in the Brillouin zone (*μ*_Γ − *X*_) for defective GaAs/AlAs superlattice

Defect type	Band gap (eV)	Effective mass (${m}_{\Gamma}^{\ast }$)	Electron mobility (*μ*_Γ − *X*_)
Ideal			
–	2.06	0.113 (0.07^a^)	0.623 (1.0^a^)
Antisite			
Ga_Al_	1.98	0.124	0.567
Al_Ga_	2.01	0.142	0.496
As_Ga_	–	0.163	0.432
As_Al_	–	0.119	0.591
Ga_As_	0.1	0.267	0.263
Al_As_	0.15	0.227	0.311
Interstitial			
Ga_int_	–	0.313	0.225
Al_int_	–	0.289	0.243
As_int_	–	0.223	0.315
Vacancy			
V_Ga_	0.47	0.729	0.097
V_Al_	0.44	0.682	0.103

*X*_*Y*_: (X, Y = Ga, Al, or As) X occupying the Y lattice site; *V*_*X*_: X vacancy; *X*_*int*_: X interstitial. ${m}_{\Gamma}^{\ast }$ in the units of the static electron mass m_e_; *μ*_Γ − *X*_ in the units of 10^4^ cm^2^/Vs. ^a^Ref. [38]

## Conclusions

In this work, a hybrid density functional theory study is performed to investigate the effects of point defect on the electrical properties of GaAs/AlAs superlattice (SL). The calculated defect formation energies show that the antisite defects are the most favorable in bulk GaAs and AlAs. In GaAs/AlAs SL structure, the antisite defects are always dominant under cation-rich and As-rich conditions and the interstitial defects are very difficult to form during the whole range of chemical potentials. It is shown that the different point defects have various effects on the electronic structures of GaAs/AlAs SL. The As_X_ (X = Al or Ga) and X_As_ antisite defects always induce metallicity, although the defective SLs with X_As_ antisites show spin splitting. As for vacancies, the defective SL with V_As_ shows metallicity character, and the group III vacancy defects reduce the band gap of the superlattice significantly. The metallicity is also found in the defective GaAs/AlAs SL with the interstitial defects. The further carrier mobility calculations show that the interstitial and vacancy defects reduce the electron mobility significantly, while the antisite defects have relatively smaller influence.

## Abbreviations

2-DEG: Two-dimensional electron gas
AIMD: *Ab initio* molecular dynamics
Al: Aluminum
AlAs: Aluminum arsenide
As: Arsenic
As_X_: As occupying the X lattice site
DFT: Density functional theory
Ga: Gallium
GaAs: Gallium arsenide
HEMT: High electron mobility transistors
HSE: Heyd-Scuseria-Emzefhof
LED: Light-emitting diode
N: Nitrogen
PDOS: Partial density of state
QCLs: Quantum cascade lasers
SL: Superlattice
VASP: Vienna *Ab Initio* Simulation Package
V_X_ (X = Ga, Al or As): X vacancy
X_As_: X occupying the As lattice site
X_int_: X interstitial
Zn: Zinc
